# Effects of polyphenolic maqui (*Aristotelia chilensis*) extract on the inhibition of NLRP3 inflammasome and activation of mast cells in a mouse model of Crohn’s disease-like colitis

**DOI:** 10.3389/fimmu.2023.1229767

**Published:** 2024-01-12

**Authors:** Tamara Ortiz-Cerda, Federico Argüelles-Arias, Laura Macías-García, Victoria Vázquez-Román, Gladys Tapia, Kangzhe Xie, María Desirée García-García, Manuel Merinero, Josefa-María García-Montes, Ana Alcudia, Paul K. Witting, Manuel De-Miguel

**Affiliations:** ^1^ Departamento de Citología e Histología Normal y Patológica, Facultad de medicina, Universidad de Sevilla, Seville, Spain; ^2^ Redox Biology Group, The Charles Perkins Centre, School of Medical Sciences, Faculty of Medicine and Health, The University of Sydney, Sydney, NSW, Australia; ^3^ Departamento de Medicina, Facultad de Medicina, Universidad de Sevilla, Seville, Spain; ^4^ Department of Gastroenterology, University Hospital Virgen Macarena, Seville, Spain; ^5^ Molecular and Clinical Pharmacology, Institute of Biomedical Sciences, Faculty of Medicine, University of Chile, Santiago, Chile; ^6^ Departamento de Química Orgánica y Farmacéutica, Universidad de Sevilla, Seville, Spain

**Keywords:** Crohn’s disease, polyphenols, anthocyanins, maqui, mast cells, NLRP3 inflammasome, interleukin 1β

## Abstract

**Introduction:**

Crohn’s disease (CD) involves activation of mast cells (MC) and NF-кB in parallel with the PPAR-α/NLRP3 inflammasome/IL-1β pathway in the inflamed colon. Whether polyphenols from maqui (*Aristotelia chilensis*) represent a natural alternative treatment for CD is unclear. Therefore, we used an animal model of 2,4,6-trinitrobenzene sulfonic acid (TNBS)-induced CD-like colitis to investigate protective effects of maqui extract through monitoring NLRP3 inflammasome and MC activation in colon tissue.

**Methods:**

Maqui extract was administered via orogastric route to mice after (post-Treatment group) or prior (pre-Treatment group) to TNBS-induction. Colon pathology was characterized by histoarchitectural imaging, disease activity index (DAI), and assessing NF-кB, p-NF-кB, PPAR-α/NLRP3 expression and IL-1β levels.

**Results:**

Compared to mice treated with TNBS alone administration of anthocyanin-rich maqui extract improved the DAI, colon histoarchitecture and reduced both colon wet-weight and transmural inflammation. Induction with TNBS significantly increased colonic NLPR3 inflammasome activation, while co-treatment with maqui extract (either post- or pre-Treatment) significantly downregulated NLRP3, ASC and caspase-1 levels, which manifested as reduced colonic IL-1β levels. Supplemented maqui extract marginally diminished NF-кB activity in epithelial cells but reached statistical significance in immune cells (as judged by decreased NF-кB phosphorylation). PPAR-α signaling was largely unaffected by Maqui whereas MC infiltration into the colon mucosa and submucosa decreased and their level of degranulation was suppressed.

**Conclusion:**

These outcomes show the post- and pre- Treatment effect of a polyphenolic extract rich in anthocyanins from maqui the acute phase of TNBS- induced CD-like colitis is linked to suppression of the NLRP3 inflammasome and reduced MC responses. These data indicate that maqui extract represents a potential nutraceutical for the treatment of inflammatory bowel disease (IBD).

## 1 Introduction

Inflammatory bowel disease (IBD) represents a cluster of chronic systemic intestinal disturbances that comprise Crohn’s disease (CD), ulcerative colitis (UC), and indeterminate colitis (CI) (1). The clinical course of IBD is characterized by unpredictable periods of cyclic remission and relapse (2). Although the precise pathogenesis of IBD is unclear, it is commonly accepted that an unregulated immune system, dysregulated intestinal microbiota, genetic predisposition, and environmental stimuli are precipitating factors (3). The immune mechanism underlying CD and UC pathogenesis is considered to be mediated by Th1 and Th2, respectively (4). The experimental mouse model using 2,4,6-trinitrobenzene sulfonic acid (TNBS) elicits a colitis-like phenotype with histopathological and morphological changes related to CD. Thus, TNBS administration stimulates a heightened Th1-Th17 response and promotes characteristic histological changes in the colon such as loss of tissue architecture, and transmural immune cell infiltration, which closely resembles important histopathological aspects of human CD (5).

Mononuclear hematopoietic and intestinal epithelial cells serve as the first line for colon defense and mediate the innate immune response stimulated by microbes or signaling through pattern recognition receptors (PRR) such as Toll-like receptors (TLRs) and nucleotide-binding oligomerization domain protein-like receptors (NLRs), or inflammasome proteins such as the NLR family pyrin domain (NLRP1, NLRP3 and NLRC4) and absent in melanoma 2 (AIM2) (6). Thus, inflammasomes are comprised of multiproteic cytoplasmic complexes that assemble via the action of the adaptor protein apoptosis-associated speck-like protein (ASC) and procaspase-1.

Activated caspase 1 cleaves pro-interleukin (IL)-1β and pro-IL-18 to yield mature pro-inflammatory IL-1β and IL-18, respectively (7). Specifically, IL-1β is primarily produced by infiltrating immune cells such as monocytes and dendritic cells as well as mature macrophages in the colon lamina propria, which explains the local and systemic effect of the IBD (8, 9). Of the proteins comprising the inflammasome, NLRP3 is the most studied receptor and considered a crucial regulator of intestinal homeostasis that is critically involved in IBD pathogenesis and disease progression (10, 11). The presence of lipopolysaccharides (LPS), UV light, and reactive oxygen species (ROS) can activate endogenous transcription factors such as nuclear factor kappa-B (NF-кB), which drive the expression of inflammatory molecules, including the components of NLRP3 (12, 13). Concomitant with transcriptional activation of NF-кB, the transcription factor peroxisome proliferator activated receptor-gamma (PPAR-α) is stimulated by microbial factors, tumor necrosis factor-α (TNF-α), LPS, and ATP (14, 15). PPAR-α is a master regulator for the transcription of target genes, inhibitor of immune cell activation and protective effects linked to inflammatory processes mediated by NF-кB (16). For example, Palenca I., et al. (2022) demonstrated that the downregulation of the TLR-4/NLRP3 pathway in experimental UC is dependent on PPAR-α activation (17). Resident colonic mast cells (MC), classified as Tryptase (MC_T_) and tryptase and chymase type (MC_TC_), can release proteases to damage the mucosal barrier and accumulate in different colonic compartments during IBD pathogenesis (18). For example, increased MC in hypertrophic and fibrotic *muscularis propria* (19) and MC accumulation in the *mucosa*, *submucosa* and *muscularis propria* is documented in patients with active CD (20). Furthermore, accumulating MC are detected in colons from CD and UC subjects with irritable bowel syndrome (IBS)-like symptoms, and subjects diagnosed with IBS compared to age-, sex-, duration and extent of disease-matched controls (21). Emerging studies have also reported that intestinal MC_T_ and NLRP3 inflammasome activation occur in parallel thereby, implicating a critical role for MC in colon inflammation (22).

Conventional treatments for patients with UC or CD have side effects with variable severity and there is no definitive cure. Therefore, the use of nutraceutical or dietary supplements such as probiotics and polyphenols may represent promising alternatives or an adjunctive to current therapies. Several studies using murine models of IBD have demonstrated that bioactive polyphenols such as epigallocatechin-3-gallate (EGCG) (23), curcumin (24) or resveratrol (25) act via anti-inflammatory and antioxidant properties, as well as impacting the gut microbiome ultimately maintaining intestinal health and colon-barrier function. Recent studies with maqui fruit (*Aristotelia chilensis*) have focused on its potential health benefits attributed primarily to anthocyanin polyphenols (26). We have shown previously that maqui fruit extract contains a high polyphenolic content and is rich in anthocyanins, especially delphinidins (27). *In vitro* studies with epithelial cells and macrophages demonstrated that maqui extract has noncytotoxic effects, and powerful antioxidant capacity which contribute to its anti-inflammatory action (27). Supplemented maqui extract significantly ameliorated the clinical course of the disease and the extent of intestinal inflammation in an experimental animal model of CD. Thus, maqui provided either post- or pre-Treatment down-regulated COX-2 and iNOS protein expression, up-regulated nuclear factor E2-related factor 2 (Nrf-2)/antioxidant heme oxygenase 1 (HO-1) signaling pathway and altered macrophage polarization towards the inflammation resolving M2 phenotype (28). Overall, the aim of this present study is to explore the anti-inflammatory effects of a polyphenolic extract rich in anthocyanins from *Aristotelia chilensis* through inhibition of NLRP3 inflammasome and MC activation using an animal model of TNBS-induced CD-like colitis.

## 2 Materials and methods

### 2.1 Polyphenolic maqui extract and treatment dosage

To obtain a large-scale quantity of maqui berry extract, the following extraction procedure was performed using acidified methanol at pH 1 (MeOH/H^+^ 0.1% v/v HCl) with the primary goal to prevent chemical changes and preserve polyphenolic components (27). Thus, all workup procedures were conducted in the dark and controlled temperature (room temperature during the collection of organic phase and 35°C during solvent evaporation). The final product was lyophilized and stored at -20°C. Where required, lyophilized polyphenolic maqui extract was dispersed in water and administered via orogastric tube (total amount 50 mg/kg/day) given in a single daily dose as per the four treatment groups outlined below.

### 2.2 Induction of acute Crohn’s disease and experimental design

This study was approved by the Ethical Committee of the Faculty of Medicine, University of Seville and the animal handling was conducted in accordance with the Guide for the Care and Use of Laboratory Animals [22]. Experimental colon disease was induced with TNBS (Sigma-Aldrich; administered at 100 mg/Kg body weight) delivered through an intracolonic path as reported previously (28).

Male Balb/c mice 12-14 weeks old were randomly assigned in 4 groups (n=6) as follows:

1) Control Group: Mice were undergoing to intra-rectally intervention using ethanol (EtOH 50%) neither TNBS nor maqui extract.
2) Crohn’s disease-like Colitis Group (CD group): 100 mg/kg of TNBS plus EtOH 50% without maqui extract.
3) post-Treatment Group: Maqui extract was administrated for 4 days after TNBS induction.
4) pre-Treatment Group: Maqui extract was administrated for 7 days prior TNBS and 4 days after TNBS induction.

### 2.3 Disease activity index and colon macroscopic evaluation

The disease activity index (DAI) score was obtained following the criteria published previously by Gommeaux et al. (29). The scoring system was used for monitoring the experimental mice from day 0 until the end of experiment to quantify disease progression. Thus, the extent of diarrhea, rectal bleeding, and weight loss were monitored daily and graded from 0 to 3 consistently by the same researcher to yield a total score that constitutes the DAI. At the end of the experiment, the average total DAI score was determined to capture the gross progression of disease. After 5 days, mice were anesthetized, the abdominal cavity was opened and the presence of adhesion and structural variations of large intestine such as ulcerations, oedema and changes in the stool consistency were documented. Subsequently, the isolated colon was rinsed with PBS and cleaned of stool to ascertain wet weight. Next, images of colonic morphology were captured using a Canon EOS 350 zoom camera (Canon Inc. Tokyo, Japan). Thereafter, the large intestine was cut in two halves longitudinally. One half was assigned to histopathological analyses and immediately fixed with 4% v/v buffered paraformaldehyde while the remaining colon was frozen in liquid nitrogen and assigned for biochemical analyses.

### 2.4 Histopathological study

For histopathological examination, the large intestine was dissected and rolled from the distal to the proximal end to form a “swiss roll”. The longitudinal fractions of the colon of each animal were fixed in 4% v/v buffered paraformaldehyde and embedded in paraffin. Thereafter, sections of tissue (4 μm thickness) were cut using a rotary microtome (Microm HM 310, Thermo Scientific), mounted on glass slides and dried (2 h, 60°C) before staining with different techniques. All histology slides were analyzed in a blinded fashion by two pathologists using an Olympus microscope (Vanox AHBT3, Tokyo, Japan) equipped with a Nikon DS-Fi3 camera.

#### 2.4.1 Hematoxylin & eosin staining

Where required, colon sections were deparaffinized, hydrated and stained with hematoxylin–eosin according to standard protocols. All stained sections were imaged, and characteristic histopathological changes were examined including inflammatory infiltrate, loss of histoarchitecture, presence of ulcerations, presence of crypt abscesses and cellular necrosis. All sections were assessed at image magnifications of 40X and 200X to conduct histology for the whole organ.

#### 2.4.2 Toluidine blue staining and quantification of mast cells

To evaluate the number and location of MC, colon tissue was stained with toluidine blue stain. Briefly, samples were immersed in a toluidine blue solution at 0.5% w/v toluidine blue (Sigma-Aldrich-Germany) in distilled water acidified with glacial acetic acid at 1% v/v for 5 min. Subsequently, sections were rapidly dehydrated with ethanol (90 and 100% each x 1 min). After two further consecutive washes in xylene (2x and 5x min), stained sections were cover-slipped using DPX mountant and visualized using an Olympus photomicroscope allowing for complete imaging from the distal to the proximal colon end. Cells detected were identified as either tryptase MC (MC_T_) or tryptase and chymase MC (MC_TC_) based on their localization in mucosa or submucosa connective tissue, respectively. Therefore, frequency and morphology characteristics including size and degranulation were evaluated. The proportion of MC subtype in each compartment was finally expressed as a percent (%) of total cell population in the relevant compartment.

### 2.5 Immunohistochemistry studies

Immunostaining for NF-kB, p-NF-kB and PPAR-α was performed in colon sections after dewaxing and rehydration followed by heat-induced antigen retrieval. During the antigen retrieval process, EDTA buffer pH 8.0 was used for NF-кB and phospho-NF-кB (pNF-кB) whereas citrate buffer pH 6.0 was used for PPAR-α. Endogenous peroxidase activity and non-specific binding was blocked with hydrogen peroxide and normal horse serum, respectively. Mouse IgG blocking with ReadyProbes™ Mouse on Mouse IgG Blocking Solution was conducted for NF-кB. Incubation with primary anti-NF-кB (Thermo Fisher Scientific Cat# PA1-30408, RRID : AB_1958961), anti-p-NF-кB (Santa Cruz Biotechnology Cat# sc-136548, RRID : AB_10610391) and anti-PPAR-α (Affinity Biosciences Cat# AF5301, RRID : AB_2837786) were performed overnight at 4°C.

Secondary antibodies were obtained from the ImmPACT DAB kit (Vector, USA) following manufacturer’s recommendations. Mayer’s Hematoxylin was used for nuclear contrast (Modified solution according to Lillie, ScyTek Laboratories, Utah, U.S.A). Analysis of positive nuclei was performed under light microscope in a blind fashion in 10 adjacent (400X magnification) per slide. The intact area and inflammatory regions were select and saved for immunostaining. Scoring of immunopositive^+^ stained sections was performed as described previously (30, 31). To evaluate NF-кB and p-NF-кB expression in epithelial cells, the total percentage of positive cells (200x magnification) were recorded within representative areas for each isolated colon and averaged across all colons in the same treatment group. For PPAR−α expression, scoring was according to the proportion of epithelial cells that stained immunopositive^+^ for PPAR−α expression determined as a percentage, yielding corresponding pathological scores of 1 for <25% of cells being stained, 2 for 25−50% and 3 for >50% cells immune^+^ for PPAR−α. The cytoplasmic expression of PPAR-α, NF-кB and p-NF-кB in macrophage cells was performed by selecting *hotspots* and manually counting cells across 10 fields (@400x magnification) per slide. Overall, the extent of pathology was scored independently by 2 blinded experts and the compiled data averaged.

### 2.6 Isolation of cytoplasmic proteins and western blot assay

Frozen colonic tissues were randomly selected (4 per group), weighed and homogenized in ice-cold buffer (containing: 50 mM Tris-HCl, pH 7.5, 8 mM MgCl_2_, 5 mM ethylene glycol-bis (2-aminoethylether)-*N,N,N´,N´-*tetraacetic acid, 0.5 mM EDTA, 0.01 mg/mL leupeptin, 0.01 mg/mL pepstatin, 0.01 mg/mL aprotinin, 1 mM phenylmethylsulfonyl fluoride (PMSF), and 250 mM NaCl). The homogenates were centrifuged at 12,000 × g, 4°C for 15 min, and the clarified supernatants were collected and stored at −80°C. Protein concentration of the colon homogenates was determined by Bradford’s method (32). Aliquots of supernatants containing equal amounts of protein (normalized to 30 μg) were separated on 10% acrylamide gel by sodium dodecyl sulfate polyacrylamide gel electrophoresis then transferred onto a nitrocellulose membrane, which was then stained with Ponceau red to confirm equal loading of total proteins. Next, the membranes were blocked with 5% BSA in Nonidet™ at 0.5% and PBS. Then, the membranes were incubated with specific primary antibodies: rabbit anti-NLRP3 (final dilution 1:1000 v/v; Cell Signaling Technology Cat# 15101, RRID : AB_2722591), rabbit anti-ASC (final dilution 1:1000 v/v; Cell Signaling Technology Cat# 70891, RRID : AB_2799791) and rabbit anti-caspase 1 (final dilution 1:1000 v/v; Abcam Cat# ab1872, RRID : AB_302644) overnight at 4°C. After rinsing, the membranes were incubated with the horseradish peroxidase-linked (HRP) secondary antibody anti-rabbit (1:1000 v/v; Cell Signaling Technology Cat# 7074, RRID : AB_2099233) Cayman Chemical, Ann Arbor, MI, USA) or anti-mouse (1:1000 v/v; Agilent Cat# P0447, RRID : AB_2617137) containing blocking solution for 1 h at room temperature. To prove equal loading, the identical blots were also analyzed for β-actin expression using an anti-β-actin antibody (1:1000 v/v; Sigma Aldrich, St. Louis, MO, USA).

All immunodetection was performed using an enhanced chemiluminescence light-detecting kit (SuperSignal West Pico Chemiluminescent Substrate, Pierce, IL, USA). Immunosignals were captured using Amersham imager 600 (Healthcare Life Sciences, Buckinghamshire, UK) and densitometric data were studied following normalization to the housekeeping protein such as β-actin used as an internal control. The signals were analyzed and quantified by an Image Processing and Analysis in Java (Image J, Softonic ®, National Institute of Mental Health, Bethesda, MD, USA) and expressed relative to the DSS-exposed control group (arbitrarily assigned as 100%).

### 2.7 Determination of IL-1 β production

Production of colonic IL-1β was determined using commercial enzyme-linked immunosorbent assay (ELISA) kits (Murine IL-1β, PeproTech, Cranbury, USA) according to the manufacturer’s protocol. Absorbance determined at 415 nm with a microplate reader (Sinergy HT, Biotek®, Bad Friedrichshall, Germany). Levels of this cytokine were determined in duplicate and expressed as picograms per milligram of wet weight tissue (pg/mg tissue).

### 2.8 Statistical analysis

All data were represented as means ± SEM and analyzed using GraphPad Prism 8.0 program (GraphPad Software, San Diego, Canada). In all cases, heterogeneity was tested by using Levene’s test and Shapiro-Wilk test for normalcy. For between group comparison of means (n=3 technical repeats), parametric data were analyzed by One-way analysis of variance (ANOVA) whereas a Kruskal-Wallis test was used for any non-parametric data sets, followed by Bonferroni’s *post hoc* test. *p-values*< 0.05, <0.01 or <0.001 were considered statistically significant.

## 3 Results

### 3.1 Treatment with maqui extract rich in Delphinidin improve clinical outcomes and macroscopic parameters in acute TNBS-induced Crohn’s disease-like colitis

**Figure 1:**
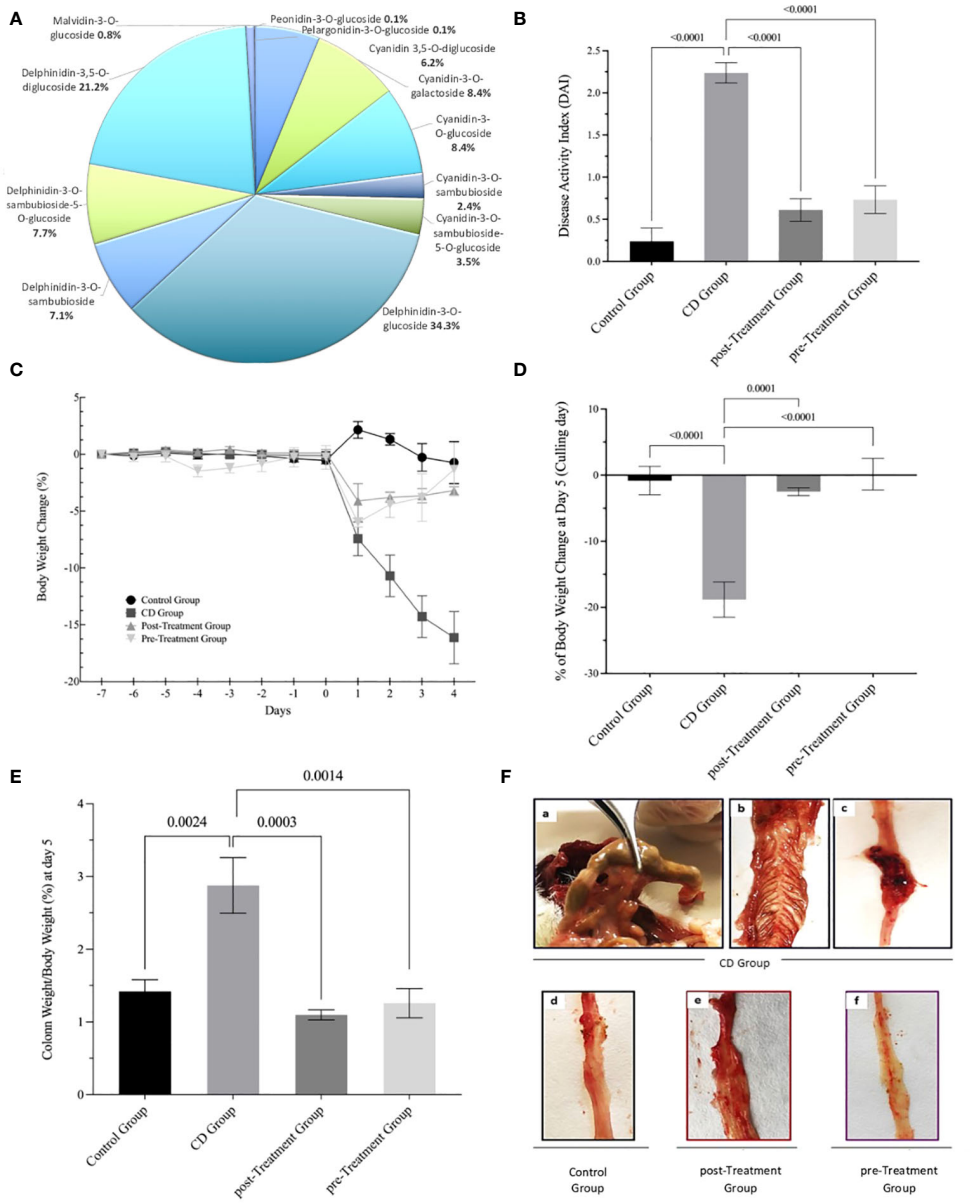
Maqui extract rich in Delphinidin prevent weight loss, improve clinical outcomes and macroscopic parameters in experimental animal model of Crohn’s disease-like colitis induced by TNBS. **(A)** Distribution of polyphenolic compounds in maqui extract as determined by (UHPLC-MS/MS) Orbitrap. The data are expressed as a proportion of polyphenol content. The effect of orogastric maqui extract on **(B)** DAI score recorded throughout the entire experiment, **(C)** Percent body weight loss in mice over the experiment and until sacrifice (28), **(D)** Percent body weight at day 5 (culling day), **(E)** Colon weight as percent of body weight on day 5 **(F)** Representative macroscopic image from the entire intact colon isolated from CD group (a,b,c), Control group (d), post- and pre-Treatment groups (f) and **Supplementary Figure S1**. Values represent mean ± SEM (*n*=6). *p-value* < 0.05, *p-value* < 0.001 and *p-value* < 0.0001; one-way ANOVA followed by Bonferroni’s Multiple Comparison test are shown between different experimental groups.

Data in **Figure 1A** shows the previously published proportion of polyphenolic compounds from the maqui extract (27). Phenolic compounds separated by UHPLCMS/MS identified high levels of Delphinidin (70.4%) and Cyanidin (28.8%), mainly comprising Delphinidin-3-*O*-glucoside 34.3% and Cyanidin-3-*O*-glucoside 8.3%, respectively, along with other anthocyanins such as Malvidin-3-*O*-glucoside (0.8%), Pelargonidin-3-*O*-glucoside (0.1%) and Peonidin-3-*O*-glucoside (0.1%). To test the effect of this maqui extract we carried out an intervention using an experimental model of CD-like colitis using TNBS to trigger a colonic inflammatory response that closely resembles immunological (33) and histopathological aspects of human CD (28). The daily clinical index increased significantly in mice exposed at TNBS at day 5. Whereas, this index was attenuated significantly in the post- and pre-Treatment groups, that typically showed diminished rectal bleeding and improved stool consistency (**Figure 1B**). As anticipated, the mice from the CD group showed a notable decline in percent body weight post TNBS administration. As previously reported (28), mice assigned to the post-and pre-Treatment groups both recorded a lower extent of weight loss at day 2 of treatment compared to mice treated with TNBS alone. Notably, mice treated with maqui extract prior to TNBS treatment showed a recovery in body weight from the point of disease induction until sacrifice (refer to reference (28) and **Figure 1C**), At the intervention end point (day 5), mice from the experimental CD group showed a significant percent body weight loss (decreasing ~19% vs the control group). By contrast, mice treated with maqui extract either by post- or pre-interventions consistently presented with less weight loss (2.5% and 0.1%, vs control, respectively) (**Figure 1D**). At day 5, a significant increase in body weight-corrected colon weight was determined (2.9%) in the experimental CD-like colitis group in the absence of maqui extract. By contrast, the increase in colon weight from mice co-treated with TNBS and maqui extract was markedly lower (1.1% and 1.3% for post- and pre-Treatment groups vs control, respectively) consistent with the natural product inhibiting tissue oedema and inflammation (**Figure 1E**). Macroscopically, extensive hyperemia, marked bowel wall thickening and adhesions between the colon and adjacent tissues was observed in colons isolated from the experimental CD-like colitis group (**Figure 1F**a, b, c and **Supplementary data S1**). All these pathological parameters were diminished by supplementation with the extract (compare images d, e, f from **Figures 1F**).

### 3.2 Maqui extract alleviates histology damage in acute experimental animal model of Crohn’s disease-like colitis

**Figure 2:**
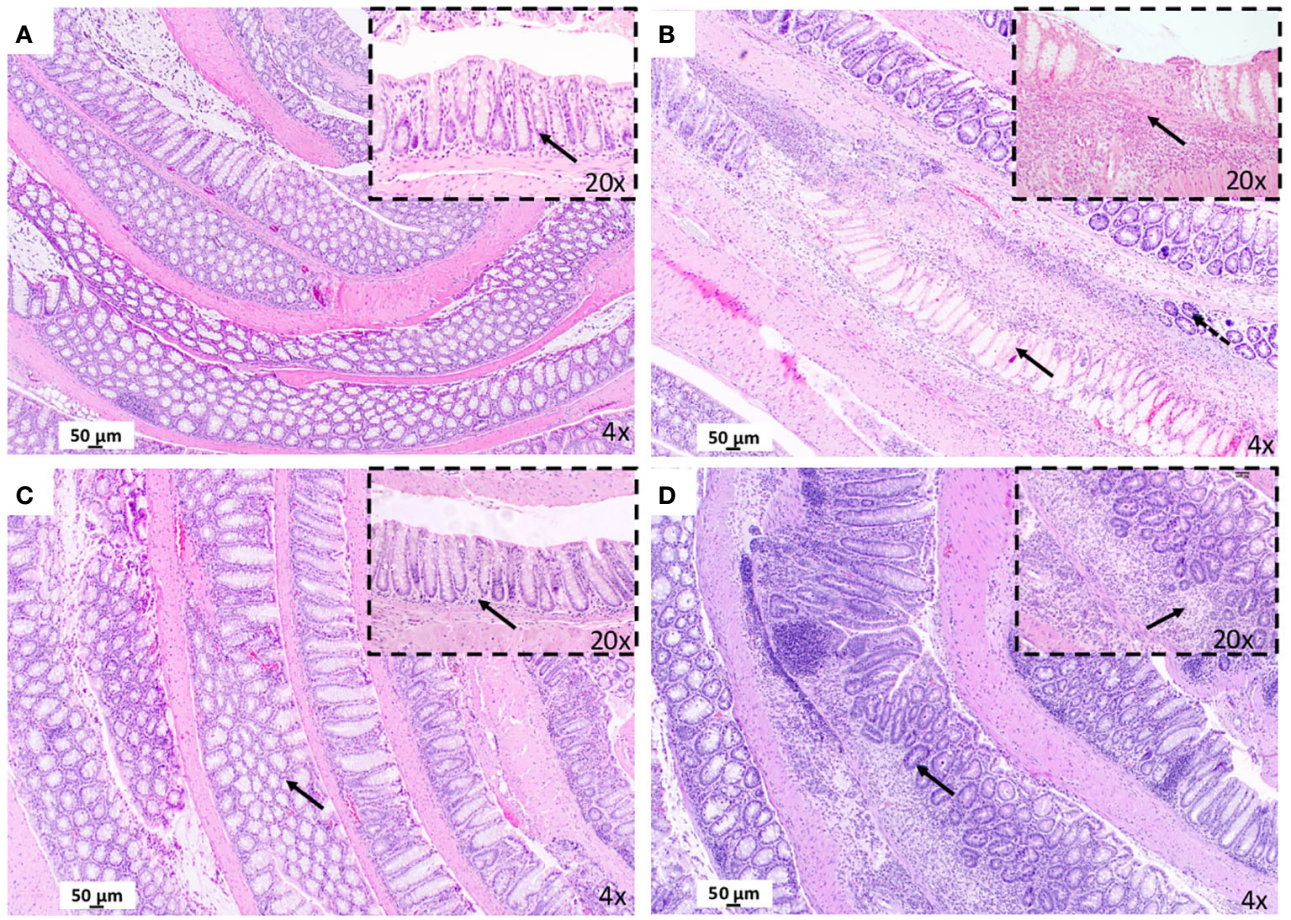
Treatment with maqui extract improve histopathological evaluation of colonic tissue from animal model of Crohn’s disease-like colitis after Hematoxylin and Eosin staining (H&E). A representative image from entire “Swiss rolled” colon evaluation from the distal to proximal end at 40x magnification (objective 4) (*n*= 6 mice/group, see **Supplementary Figure S2**). **(A)** Control Group: Normal histology colon tissue. Physiology immune cells in lamina propria (black arrow in 10x). **(B)** CD Group: Presence of necrosis (black arrow in 40x). and goblet cells depletion (dashed arrow). Transmural infiltrate inflammatory (black arrow in 20x). **(C)** post-Treatment group: Preservation of the colonic mucosa structure (black arrow in 40x). Normal cells infiltration (black arrow in 20x). **(D)** pre-Treatment group: Lesser extent of atrophy (black arrow in 4x) and mucosal crypts loss (black arrow in 20x).

Severe and extensive transmural infiltration of neutrophils and eosinophils was detected in the colon mucosa and submucosa from mice with experimental CD-like colitis relative to the control group and in the absence of maqui (compare **Figures 2A, B** and **Supplementary S2**). This extensive infiltration was accompanied by a marked distortion of colon architecture, with evidence of focal necrosis and goblet cells depletion. By contrast, colons from mice co-treated with TNBS and maqui extract were consistently protected from inflammation as evidenced by a decrease in inflammatory cell infiltration and recovery of intestinal architecture (**Figure 2C**). Atrophic mucosa and loss of cytoplasm were still observed in mice from the post-Treatment group albeit at lower extents of focal glandular atrophy and mucosal crypts loss. Therefore, signs of chronic inflammation remained in these tissues (**Figure 2D**).

### 3.3 Maqui extract reduced the activation of inflammasome NLRP3 and decrease the colon IL-1β level in the colons of mice with acute TNBS-induced Crohn’s disease-like colitis

**Figure 3:**
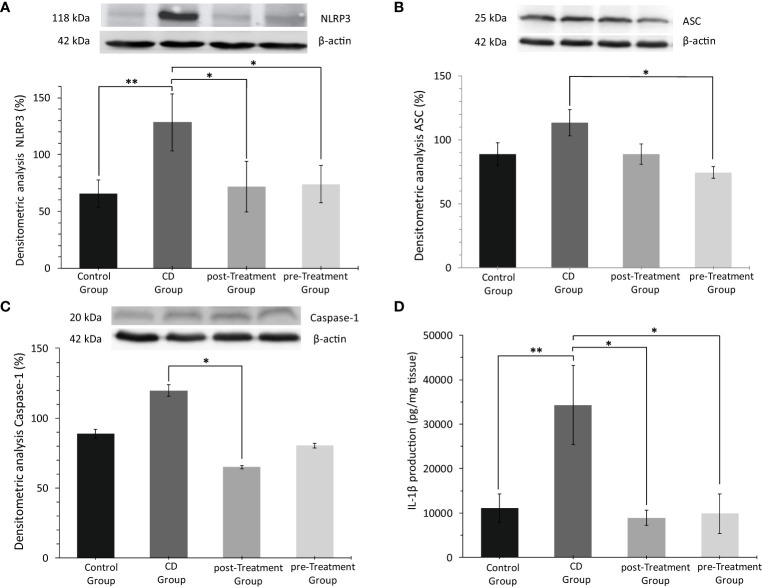
Anti-inflammatory effect of the maqui extract (50 mg/kg/d) on inhibition of multiprotein complex inflammasome NLPR3 and pro-inflammatory cytokine IL-1β from colons of Crohn’s disease-like colitis (*n*= 6 mice/group). Representative Western blot images and densitometric analysis of each group: **(A)** NLR Family pyrin domain 3 (NLRP3), **(B)** Adaptor protein apoptosis-associated speck-like protein (ASC) and, **(C)** Caspase 1. β‐actin was used as an equal loading control for normalization. **(D)** ELISA quantification of IL-1β production. Values represent mean ± SEM; *Difference at the p < 0.05 level, **Difference at the p < 0.001 level; one-way ANOVA followed by Bonferroni’s Multiple Comparison test.

An aberrant activation of NLRP3 inflammasome and an overregulation of cytokines IL-1β and IL-18 have been reported to be essential in the early phase of the inflammatory cascade in IBD (34). Data obtained here showed that TNBS stimulation in the absence of maqui markedly increased colon NLRP3 levels, while co-treatment with maqui extract, both as post- and pre-Treatment groups significantly downregulated NLRP3 levels in mice (**Figure 3A**). As indicated in **Figures 3B, C**, protein expression of ASC and Caspase 1 in the colon tissue from the experimental CD group trended to increase, although this was not significantly different to the control group. Overall, treatment with 50 mg/kg/d of maqui extract suppressed the expression of these proteins. Similarly, IL-1β levels increased significantly in mice with experimental CD-like colitis. In contrast, colon IL-1β was significantly reduced in the post- and pre-Treatment groups (**Figure 3D**). These data showed that maqui extract actively inhibited components of multiprotein complex associated with NLPR3 inflammasome activation, and this was linked to decreased IL-1β levels in the same colons, suggesting that in addition to acting via antioxidant mechanisms, this natural product also provides an anti-inflammatory effect.

### 3.4 Maqui extract treatment weakly modulates the transcriptional factor NF-кB through diminishing NF-кB phosphorylation in colon epithelial cells

**Figure 4:**
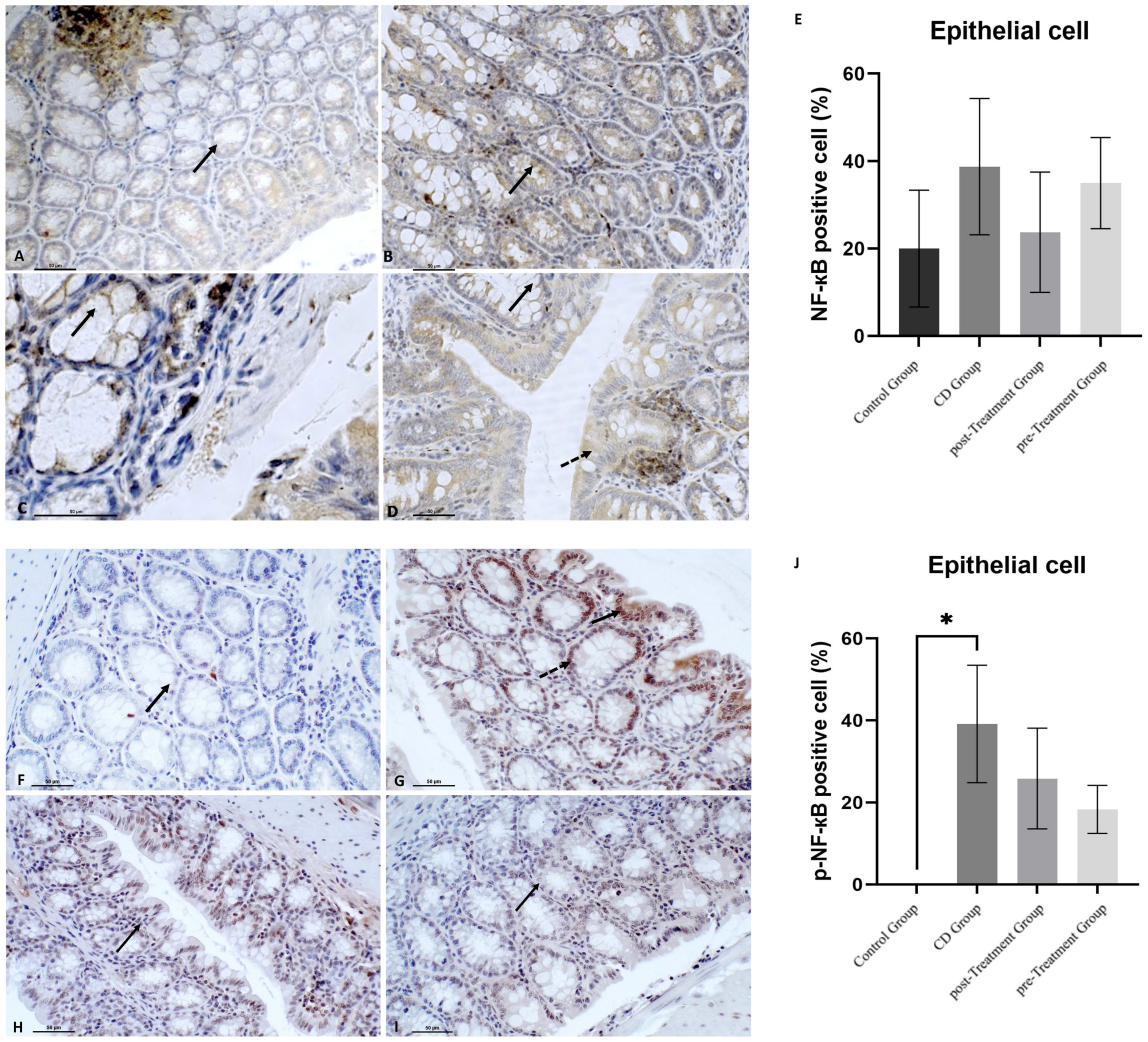
Polyphenolic maqui extract shows preventing trend of decreasing NF-кB phosphorylation in colon epithelial cells from experimental animal model of Crohn’s disease-like colitis. Immuno‐histochemistry for NF-кB in colon from all groups (*n*= 6 mice/group): Immune^+^-NF-кB staining intensity in cytoplasm from crypts cells (black arrow) in **(A)** control group, **(B)** CD group, **(C)** post-Treatment group and **(D)** pre-Treatment group **(D)**. Dashed arrow shows positive staining in colon epithelium in the pre-Treatment group (image D). Immuno‐histochemistry for p-NF-кB in colon from all groups: **(F)** Absence of staining of p-NF-кB in colons from control group (black arrow shows epithelial cell nucleus without positive staining for p-NF-кB), **(G)** Immune staining of p-NF-кB in crypts cells (black arrow) and in the epithelium (dashed arrow) in CD group, **(H)** Immune p-NF-кB staining intensity in cytoplasm from colon epithelium (black arrow) in the post-Treatment group **(I)** Immune p-NF-кB staining intensity in cytoplasm from crypts cells (black arrow) in the pre-Treatment group**. (E, J)** Percentage of positive cells (200x magnification) within representative areas for NF-кB and p-NF-кB expression in epithelial cells, respectively. Values represent mean ± SEM; *Different to the Control Group; p < 0.05; one-way ANOVA followed by Bonferroni’s Multiple Comparison test.

The transcription factor NF-кB plays a key role in mediating IBD pathologies. Once phosphorylated, NF-кB translocates from the cytoplasm to the nucleus, which subsequently stimulates NRLP3 and initiates production and secretion of proinflammatory cytokines IL-1β and IL-18, which contribute to the aberrant immune response observed in IBD (35). Data shown in **Figures 4A, B** indicated that TNBS induction resulted in more intense immune^+^-NF-кB staining and yielded an increased proportion of NF-кB positive epithelial cells compared to the control group (reaching 1.93-fold increase in mean levels), although this was not statistically significant (**Figure 4E**). After treatment with maqui extract, both post- and pre-Treatment groups, showed a weak trend to down-regulated immune^+^-NF-кB staining and intensity of the cytosolic NF-кB staining in epithelial cells (**Figures 4C, D**), which was not statistically significant (**Figure 4E**). In terms of p-NF-кB expression in the epithelial cells, there was no positive staining of p-NF-кB observed in the colons from the control group (**Figure 4F**). By contrast, an intense nuclear staining of p-NF-кB and significantly higher proportion of epithelial cell p-NF-кB positive expression was detected in colons from the experimental CD group (**Figures 4G, J**). Decreased p-NF-кB positive expression and intensity was observed in the groups that received maqui extract treatment, although this trend was not significant (compare **Figures 4H-J**).

### 3.5 Modulation of maqui extract on transcriptional factor NF-кB through the prevention of NF-кB phosphorylation in autoimmune cells

**Figure 5:**
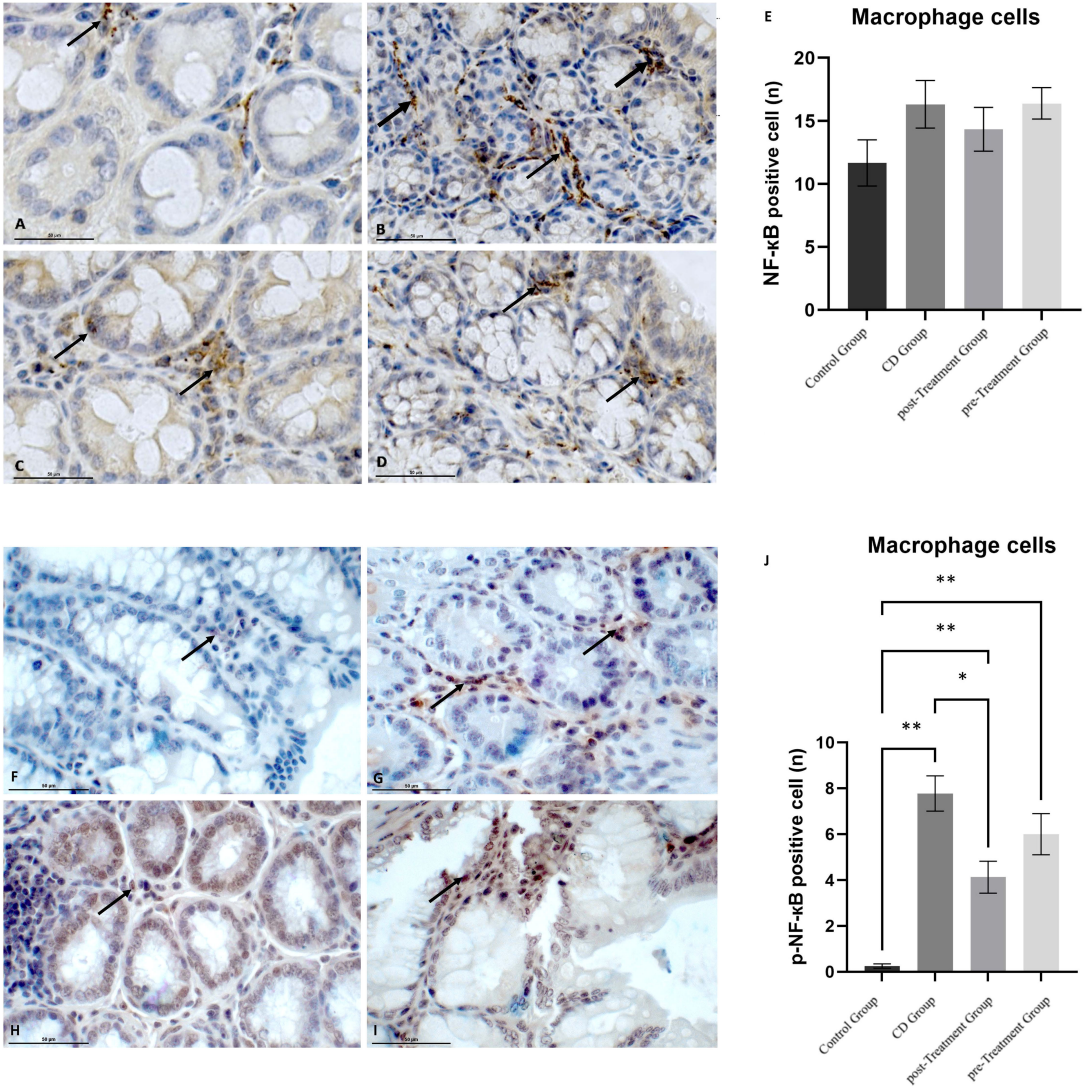
Effect of polyphenolic maqui extract in preventing NF-кB phosphorylation in macrophages cells in lamina propria from colons of Crohn’s disease-like colitis model. Immuno‐histochemistry analyses for NF-кB in 10 representative fields from colon tissue of each group (*n*= 6 mice/group): **(A)** Immune^+^-NF-кB staining intensity in macrophages cells (black arrow) in the control group, **(B)** CD group, **(C)** post-Treatment and **(D)** pre-Treatment groups. Immuno‐histochemistry analyses for p-NF-кB from 10 representative fields of view for each group: **(F)** Immune expression of p-NF-кB in macrophages cells in the control group (black arrow shows a single site in the field), **(G)** CD group, **(H)** post-Treatment group and **(I)** pre-Treatment group. Black arrows show positive staining in different sites in image **(G–I). (E, J)** Counting cells across 10 fields (400x magnification) for NF-кB and p-NF-кB expression in macrophages cells, respectively. Values represent mean ± SEM; *Difference at the p < 0.0005 level, ** Difference at the p < 0.0001 level; one-way ANOVA followed by Bonferroni’s Multiple Comparison test.

The cellular expression of NF-кB and p-NF-кB in macrophages was also examined. Similar to the trend observed in the epithelial cells, TNBS-induced experimental CD-like colitis resulted in a greater cytoplasmic intensity and number of NF-кB positive cells when compared to the control group, although again this was not significant (**Figures 5A, B, E**). The administration of maqui extract caused a weaker cytoplasmic staining intensity of NF-кB and a marginal reduction in the number of NF-кB positive cells in the post-Treatment group (**Figure 5C**). In the pre-Treatment group, maqui extract treatment did not alter the staining intensity of NF-кB nor the number of NF-кB positive macrophages (**Figure 5D**). None of these changes were statistically significant (**Figure 5E**). Data shown in **Figures 5F, G** indicate that the nuclear staining of p-NF-кB was scarce and almost no p-NF-кB positive macrophages were observed in the control mice, whereas the administration of TNBS led to strong nuclear staining of p-NF-кB and significantly increased numbers of p-NF-кB positive macrophages in the experimental CD-like disease group (**Figure 5J**). Under these inflammatory conditions, treatment with maqui extract alleviated the TNBS-mediated increase in p-NF-кB levels, as judged by the weaker nuclear p-NF-кB staining intensity and number of p-NF-кB positive macrophages cells, which reached statistical significance in the post-Treatment group (**Figures 5H, I**). However, this diminution of p-NF-кB positive cells remained above levels determined in control mice in the absence of TNBS (**Figure 5J**). Together, these results suggest that Maqui extract modulates the proinflammatory NF-кB transcription activation by inhibiting the extent of phosphorylation during the inflammation induced by TNBS in mice, with a more prominent effect observed in the post-Treatment group.

### 3.6 Potential anti-inflammatory effect of maqui extract on PPAR-α expression in epithelial and macrophage cells in colon tissue from experimental model of Crohn’s disease-like colitis.

**Figure 6:**
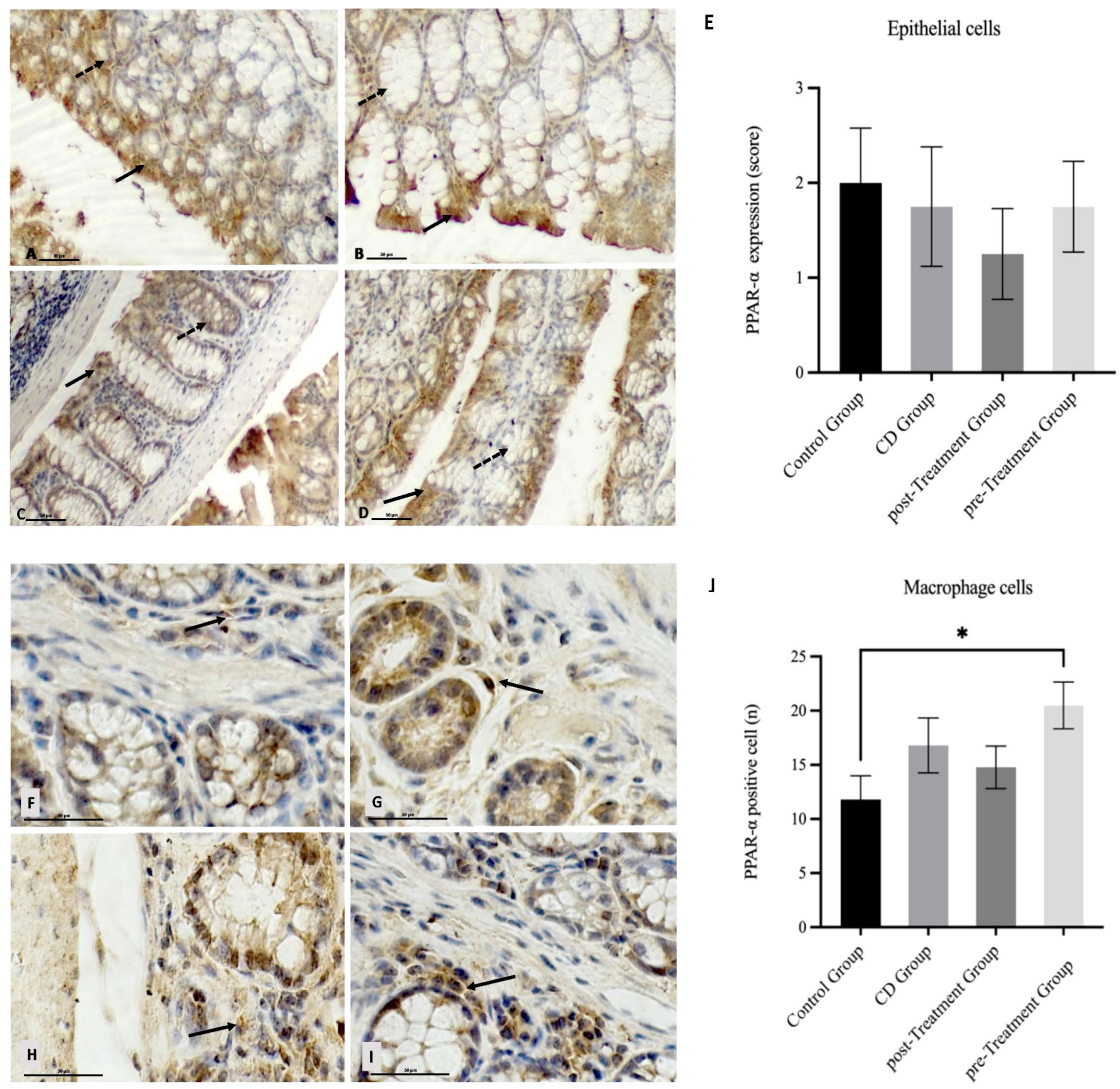
Impact of Maqui extract rich in anthocyanins on PPAR-α expression in colon tissue from experimental animal model of Crohn’s disease-like colitis. Immuno‐histochemistry analyses for PPAR-α in epithelial cells from colons of each group (*n*= 6 mice/group): Positive PPAR-α staining in colon epithelium (black arrow) and crypts cells (dashed arrow) in **(A)** control group, **(B)** CD group, **(C)** post-Treatment and **(D)** pre-Treatment groups. Immuno‐histochemistry analyses for PPAR-α in macrophages cells from colons tissue from 10 representative fields of view for each group: Immune^+^-PPAR-α staining intensity (black arrow) in **(F)** control group, **(G)** CD group, **(H)** post-Treatment group and **(I)** pre-Treatment group, **(E)** Immune positive score (1= <25%; 2 = 25−50%; 3= >50%) for PPAR−α expression in epithelial cells. **(J)** Counting cells across 10 fields (400x magnification) for PPAR−α expression in macrophages cells. Values represent mean ± SEM; *Difference at the p < 0.0005 level; Kruskal–Wallis test.

It has been shown that PPAR-α exerts its anti-inflammatory action by negatively influencing the transcriptional activity of NF-кB, and in the absence of PPAR-α gene, IBD pathologies were exacerbated (16). Next, we explored whether maqui extract modulated PPAR-α expression in colon tissue. In the epithelial cells, TNBS induction resulted in a weaker PPAR-α expression score in the experimental CD-like disease group when compared to the control group (**Figures 6A, B**). Notably, this difference did not reach statistical significance (**Figure 6E**). The administration of maqui extract resulted in similar PPAR-α staining intensity and PPAR-α expression score for both post- and pre-Treatment groups compared to the corresponding control (**Figures 6C-E**).

In terms of specific macrophage PPAR-α level, a stronger staining intensity and a trend towards elevated PPAR-α positive cells were observed in the CD mice when compared to the controls however, this difference was not significant (**Figures 6F, G, J**). Similarly, an increase in PPAR-α staining intensity and PPAR-α positive macrophages were also observed in mice that received maqui post-induction of the disease (**Figures 6H, J**), whereas colons from mice receiving maqui extract as a pre-Treatment yielded a strong PPAR-α staining and significantly higher PPAR-α positive macrophages when compared to the controls (**Figures 6I, J**). These data show that during acute inflammatory processes there is upregulated expression of anti-inflammatory transcription factor in the mouse colon, likely acting as a compensatory mechanism. Also, these outcomes supported the idea that the administration of maqui extract – especially prior to TNBS induction – can potentiate macrophage expression of PPAR-α transcription factor compared to normal physiological conditions and, elicit a weak trend to increase PPAR-α expression in CD-like disease. This may suggest an anti-inflammatory role through regulating PPAR-α activation.

### 3.7 Maqui extract exert suppressed mast cells activation in the colons of mice with TNBS-induced Crohn’s disease-like colitis

**Figure 7:**
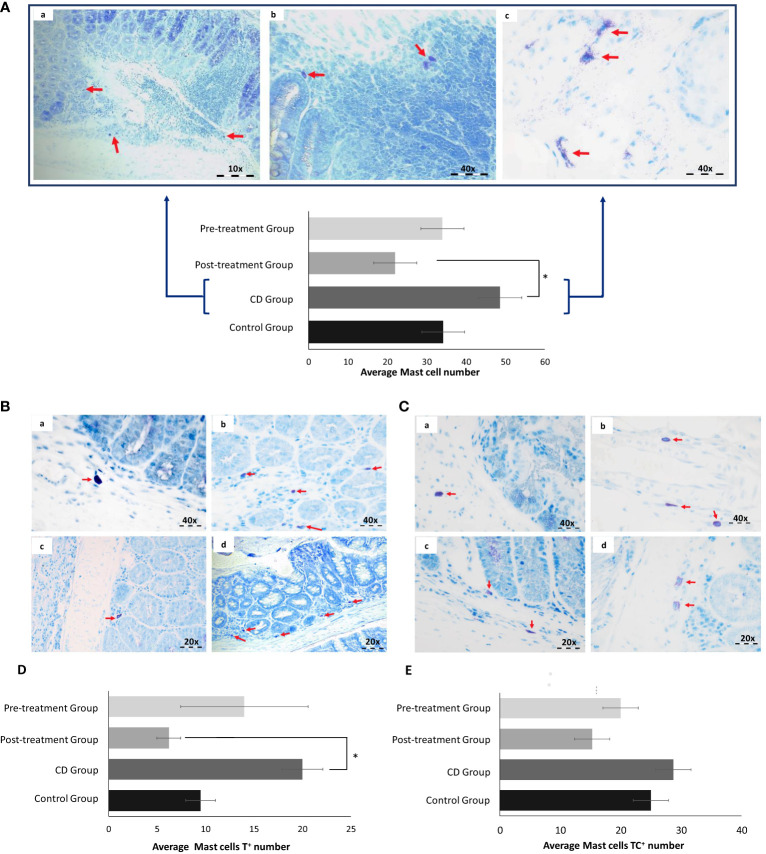
Maqui extract inhibited Mast cells (MC) activation in the experimental model of Crohn’s disease-like colitis (*n*= 6 mice/group). Toluidine blue staining and Quantification of MC. **(A)** Representative histological outcomes for CD group. Red arrows shown identify (a) small sized MC, (b) MC present in lamina propria and **(c)** degranulated MC. **(B)** Tryptase (MC_T_) and **(C)** Tryptase and chymase (MC_TC_) are shown in all groups. The red arrows show the difference in number, size, and degranulation of MC between control (a), CD (b), post-Treatment (c) and pre-Treatment groups (d). Quantification of cellular content shown as mean level of **(D)** T^+^ MC located in intestinal mucosa of each group and **(E)** TC^+^ MC located in intestinal submucosa of each group. The data shown Values represent mean ± SEM; *Difference at the p < 0.05 level. One-way ANOVA followed by Bonferroni’s Multiple Comparison test.

MC are relevant in the intestinal inflammation, however their precise role and mechanism of action during IBD pathogenesis is still unclear. To explore whether therapeutic effect of maqui extract extends to regulating MC populations in the colon, we conducted toluidine blue staining due its metachromasia properties. An increase in MC number was identified in the colons from the experimental CD group (CD; 49 ± 9.7 vs Control; 34 ± 9). The MC count in colon tissue from the pre-Treatment group was similar to the control (35 ± 13.9) and even in the post-Treatment group the total number of MC was lower than the corresponding controls (22 ± 1.2 vs 34 ± 9, respectively); albeit this failed to reach significance (**Figure 7A**).

Based on general morphological characteristics, we determined that MC in the CD group presented with relatively small size and appeared to be degranulated in the surrounding inflammatory aggregates (7Aa, b and c). Next, we identified the two main types of MC by examining their histological location, considering that MC_T_ are typically located in intestinal mucosa while MC that contain Tryptase and chymase (MC_TC_) reside primarily in the connective tissue within the intestinal submucosa. Applying these points of demarcation demonstrated that counts for MC_T_ increased in colon tissue from mice exposed to TNBS in the absence of maqui. Co-treatment with TNBS and maqui extract reduced tryptase expression, reaching significance in post-Treatment group (**Figures 7B, D**). Although we did not find significant differences in the counts for MC_TC_, the groups treated with maqui extract prior to and after TNBS-induction showed a tendency to decrease MC_TC_ count compared to the TNBS group (**Figures 7C, E**). Together, these results suggested a relationship between the increase in MC population and acute inflammation in CD. Furthermore, our data demonstrated that post- and pre-Treatment with maqui extract may prevent the granulation of MC and decrease the role for these subtypes of MC in promoting intestinal inflammation.

## 4 Discussion

The pathogenesis of CD constitutes an autoimmune disorder characterized by chronic inflammation of the gastrointestinal tract where activation of MC and infiltration of neutrophils and eosinophils represents the first immune response that triggers downstream systemic inflammation (36). Conventional therapies for CD offer limited efficacy, carry severe side effects without offering a permanent remission (37). Polyphenols are common plant-derived compounds with potent antioxidant and anti-inflammatory activities. Given the promising bioactivity of polyphenols, the utilization of naturally occurring polyphenols as a novel therapeutic strategy in IBD as well as various autoimmune conditions has gained substantial traction in recent years (38, 39). Indeed, the study by Guo X. et al. has demonstrated that curcumin reduced clinical manifestations and intestinal inflammation in DSS-induced colitis mice (40). Additionally, the polyphenol (especially anthocyanins)-rich extract of a berry originating from the Chilean Patagonia, maqui, actively downregulates proinflammatory proteins, shifts macrophage polarization towards resolution and activates the Nrf-2/HO-1 antioxidant response in a TNBS-induced model of CD-like colitis, suggesting the therapeutic potential of using natural polyphenols as a pharmaceutical intervention for IBD (28). In the present study, post- and pre-Treatment with maqui extract significantly improved the DAI and alleviated macroscopic markers of colon inflammation in the model of CD-like colitis. Importantly, results from the current study also revealed, for the first time, maqui exerts its anti-inflammatory action through diminishing activation of the transcription factor NF-кB (via inhibiting NF-кB phosphorylation) and trending to promote PPAR-α expression/activity. This activity may involve inhibition of cellular ROS known to activate the non-canonical NF-кB pathway promoting immune response(s) and inducing inflammation through upregulation of specific target genes such as cytokines and proinflammatory enzymes (41). Overall, the bioactivity for maqui polyphenols or their metabolic derivatives in the colon may be explained by a combination of enhanced antioxidant capacity and anti-inflammatory actions through the combine bioactivity of decreasing ROS production (assessed previously by DCFH oxidation (27)) and inhibiting NF-кB activation.

Several studies have demonstrated the effect of intracolonic injection of the hapten reagent TNBS to induce CD-like colitis. For example, Gessala R. et al. identified the presence of fibrosis and stenosis in rats exposed to TNBS in regions of colon inflammation and significant mechanical distention in the proximal segments, a feature commonly encountered in patients with CD (42). In the present study we detected, patchy areas of marked transmural inflammation, and severe oedema surrounding crypts with necrosis and architectural distortion in the colonic tissue. Taken together, these data indicate that the use of TNBS can represent a useful experimental approach to explore the pathophysiology mechanisms of colon inflammation, especially considering that intrarectal infusion of TNBS promotes a Th1 cell–mediated immune response that yields a colitis-like phenotype that resembles human CD (43).

Although mice cotreated with TNBS and maqui extract still showed slight mucosal atrophy, represented by loss of cytoplasm and glands, the same colons showed several foci of epithelial regeneration linked to a decrease in the content of mucosal infiltrating inflammatory cells, indicating that supplemented maqui exerted was able to exert both a protective and post-Treatment effect. Recent studies had demonstrated that anthocyanins from maqui or other sources such as strawberry (44), blueberry (45) can reduce histological damage and inflammation. For example, Gao Z. et al. reported that the ethyl acetate fraction of extract maqui exhibited a high content of active polyphenols and corresponding antioxidant capacity, and when administered at 200 mg per kg body weight concentration significantly inhibited disease progression in an experimental model of UC (46). Therefore, our study reinforces the therapeutic potential for maqui extract (50 mg per kg body weight; rich in anthocyanins) on the inflamed colonic mucosa and submucosa. Transcription and oligomerization of the NLRP3 inflammasome has been broadly linked to innate immune signaling. Negative regulators such as GTP-binding protein or CARD8 have been strongly associated with a protective function in CD through inhibition of the inflammasome NLRP3 complex assembly (47), meanwhile other factors such as oxidative species and LPS act as a strong signal to induce NF-кB activation, which, in turn, activate IL-1β and IL-18 and their release into the inflamed colon. In the current study, the experimental model of CD-like colitis has a high expression of proinflammatory cytokines, demonstrating that inflammasome NLRP3 pathway may have a relevant role in the inflammatory process. Here, we showed that the orogastric administration of polyphenols from maqui both prophylactic and therapeutic effectively suppressed the production of IL-1β through the inhibition of the NLRP3 inflammasome. In agreement with our results, Duo X. et al. reported marked colon inflammation mediated by an upregulation of NLRP3-caspase-1/IL-1β signaling pathway in another experimental animal model of colitis (48). This outcome is further supported by other research that demonstrated administration of anti-IL-β normalizes severe experimental colitis (49). Furthermore, previous studies have indicated that polyphenols can exert therapeutic effects against oxidative stress and inflammation. However, these effects were determined with higher concentration of polyphenol than tested here, indicating that the bioactivity of maqui berry has a capacity to alleviate damage induced by intestinal inflammation at doses that are lower than that required for a marked enhancement of antioxidant activity. Here, the administration of maqui extract reduced the extent of TNBS-induced NF-кB phosphorylation. This is consistent with other studies that utilized other polyphenolic compounds in inflammatory diseases. For example, administration of naturally derived polyphenol chlorogenic acid reduced neuroinflammation by inhibiting NF-кB activation as well as diminishing IL-1β and TNF-α secretion in a MPTP-mouse model of Parkinson’s disease (50). More recently, Varthya et al. demonstrated that another polyphenol-rich nutraceutical, green tea, alleviates CD pathologies and gut inflammation by inhibiting NF-кB phosphorylation in a TNBS-induced mouse model of colitis (51). During the onset of IBD, internal stimuli cause rapid activation of the canonical NF-kB pathway, which triggers the release proinflammatory cytokines IL-1β and TNF-α (52).

PPAR-α is an anti-inflammatory transcription factor that negatively influences the transcriptional activity of NF-кB (53). It has been shown that PPAR-α activation increases the expression of IкB-α and prevents the translocation of p50/p65 in the cell nucleus, and the absence of PPAR-α gene resulted in the augmentation of IBD pathologies (16). The result from our study showed that PPAR-α expression trended to increase in response to maqui treatment, which suggests that this nutraceutical potentially limits NF-кB phosphorylation by inhibiting its transcriptional activation via the canonical pathway. However, it should be noted that NF-кB activation could also be achieved through the non-canonical pathway, in which the presence of ROS induces the activation of p100 protein via the tumor necrosis factor superfamily receptors (54). Given the strong antioxidant activity of the extract as demonstrated previously (27), it is possible that maqui also inhibits the NF-кB phosphorylation through quenching cell signaling levels of ROS in the system.

Notably, in the current study two different approaches were proposed, post- and pre-Treatment. Here, a lower level of NF-кB phosphorylation was detected in the post-Treatment group whereas the pre-Treatment group displayed greater increase of PPAR-α. Thus, there is a possibility that the anti-inflammatory action of maqui was elicited through two different modes: by increasing the expression of PPAR-α and preventing the canonical activation of the NF-кB during the earlier phase of the disease, and by attenuating ROS and inhibiting non-canonical NF-кB phosphorylation during the latter phase of the disease. Nevertheless, the proportion in which these polyphenols inhibit NF-кB through the canonical and non-canonical pathways remains unclear and warrants further investigation. NF-кB also plays an integral role during the priming step of NLRP3 inflammasome activation (13). Maqui extract presents therapeutic potential through mitigating the NLRP3-driven inflammatory responses. This notion is supported by studies that utilized other polyphenol-rich nutraceuticals. For example, Wang et al. highlighted that green tea extract simultaneously reduced NF-кB signaling and NLRP3 activation in a mouse model of lipopolysaccharide-induced inflammatory liver injury (55), whilst Gong et al. demonstrated that curcumins alleviated DSS-induced colitis via inhibiting NLRP3 inflammasome activation and IL-1β production (56).

**Figure 8:**
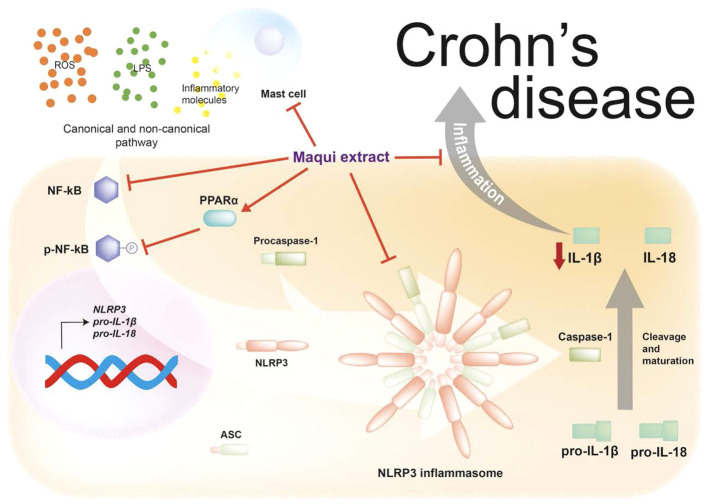
Maqui extract rich in anthocyanins inhibits the NLRP3 inflammasome components and reduces the activation of Mast cells (MC) in an acute phase animal model of Crohn’s disease-like colitis. Maqui extract reduce the activation and phosphorylation of NF-кB and upregulated the PPAR-α expression which drives inhibition of components of NLPR3 inflammasome and the subsequent decrease of IL-1β production. Additionally, Maqui extract prevents the activation and degranulation of MC, which in turn could downregulate NLRP3 inflammasome activation.

The immune response of the chronic intestinal inflammation is linked to the involvement of MC. In this context, Liu B. et al. demonstrated an increase of MC infiltration and overexpression of tryptase, a protein stored in MC granules, in IBD-Induced intestinal fibrosis (57). Interestingly, we found here that maqui extract suppressed MC activation in the inflamed colon as judged by the general small size of the cells where present in the colon and inhibited extent of degranulation in the inflamed colon isolated from mice receiving maqui extract vs TNBS injury alone. Consistent with these observations, other natural sources rich in polyphenols have been reported to decrease gut-derived MC and prevent their degranulation, resulting in the restoration of intestinal barrier function (22). Similarly, other well-known antioxidants such as vitamin E, specifically tocotrienol, also suppresses degranulation of MC via inhibiting PKC activity and preventing accumulation of MC in other inflammatory disorders such as allergic dermatitis or acute liver damage (58, 59). Furthermore, available evidence supports that MC enhance inflammasome complex formation and express components critical for the activation of caspase-1 and IL-1β secretion and are target of IL-1β pro-inflammatory signaling in the presence of proinflammatory stimuli (60). These available data potentially indicate that the preventive and therapeutic effect of maqui extract at least in part involves engagement with both MC and NLRP3 inflammasome components to modulate expression and activation. **Figure 8** shows a hypothesized mechanism of actions of maqui extract on inflammatory signaling linked to inflammasome NLRP3 and MC activation. In summary, we explored the therapeutic effects of a polyphenolic extract rich in anthocyanins from *Aristotelia chilensis* on clinical and histologic parameters and its anti-inflammatory potential effect on experimental animal model of CD-like colitis. The present study provided strong evidence that the physiological route (orogastric) administration with maqui extract can prevent or reverse the macroscopic pathology and damage elicited by intestinal inflammation. Herein, we showed the underlying mechanism whereby this potential nutraceutical can effectively present an anti-inflammatory effect. Outcomes determined here demonstrate that maqui extract most likely acts to diminish NF-кB phosphorylation with a parallel inhibition of components of NLPR3 inflammasome activation and the release of IL-1β. In the experimental model tested here, the higher expression of PPAR-α in macrophage cells from mice treated with maqui extract, especially evident in the pre-Treatment group, might ameliorate colon inflammation possibly through NF-кB-dependent or -independent pathways. Recent data suggested that pharmacological PPAR-α agonists can reduce the extent of immune cell infiltration in a murine model of airway inflammation and specifically inhibit inflammation in the airway epithelial cell, however this effect is not related to the NF-кB pathway (61). On the other hand, the use of a potent PPAR-α agonist is reported to alleviate inflammation and tissue injury during chronic inflammatory disease through a NF-кB- dependent pathway (62). Nevertheless, while we have identified that the NLRP3-inflamamsome can play a role in CD-like colitis, further studies are necessary to confirm linkages between NF-kB and PPAR-α and their influence on inflammasome NLRP3 activation targeted at pharmacologic blockade NF-kB and PPAR-α pathways in relation to the potential anti-inflammatory effect of maqui extract. Finally, the bioactive components from maqui reduced the extent of MC infiltration into the mucosa and submucosa and suppressed their degranulation, suggesting multiple beneficial activities for this nutraceutical. These datasets are consistent with a therapeutic effect of maqui extract in the acute phase of CD pathogenesis.

## Data availability statement

The original contributions presented in the study are included in the article/**Supplementary Material**. Further inquiries can be directed to the corresponding authors.

## Ethics statement

The animal study was approved by Ethical Committee of the Faculty of Medicine, Universidad de Sevilla through the Consejería de agricultura, pesca y desarrollo rural, Junta de Andalucía government. The study was conducted in accordance with the local legislation and institutional requirements.

## Author contributions

TO-C, J-MG-G-M and MD-M contributed to conception and design of the study. TO-C, LM-G, VV-R, GT, MG-G, MM and AA performed experiments, analyzed, and interpreted the data. TO-C, KX, VV-R and LM-G create and organized the database. TO-C and VV-R performed the database. TO-C prepared the first draft of the manuscript. TO-C, J-MG-G-M and KX wrote sections of the manuscripts. TO-C, MG-G and MM designed the abstract figure. FA-A, MD-M and PW edited and revised the manuscript and provided scientific input. All authors contributed to the article and approved the submitted version.
