# Supplementary material for: Effects of polyphenolic maqui (Aristotelia chilensis) extract on the inhibition of NLRP3 inflammasome and activation of mast cells in a mouse model of Crohn’s disease-like colitis
Source: Front Immunol. 2024 Jan 12;14:1229767. doi: 10.3389/fimmu.2023.1229767 (PMC10811055; doi:10.3389/fimmu.2023.1229767)
Supplement: Supplementary file 1 [file DataSheet_1.pdf]

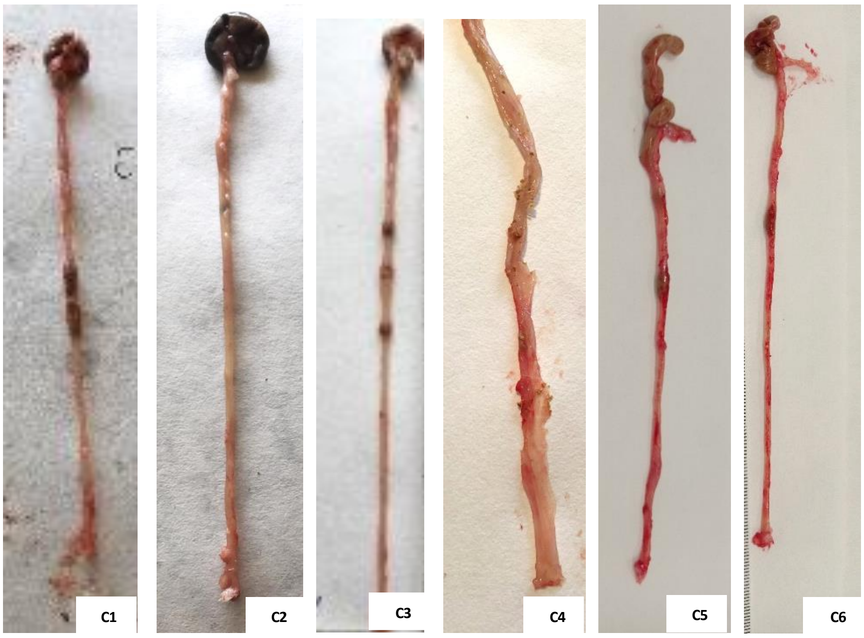

Control (C) group

Colon images from control group (C) of each mouse. The images show regular length of colon and absence of macroscopic damage.

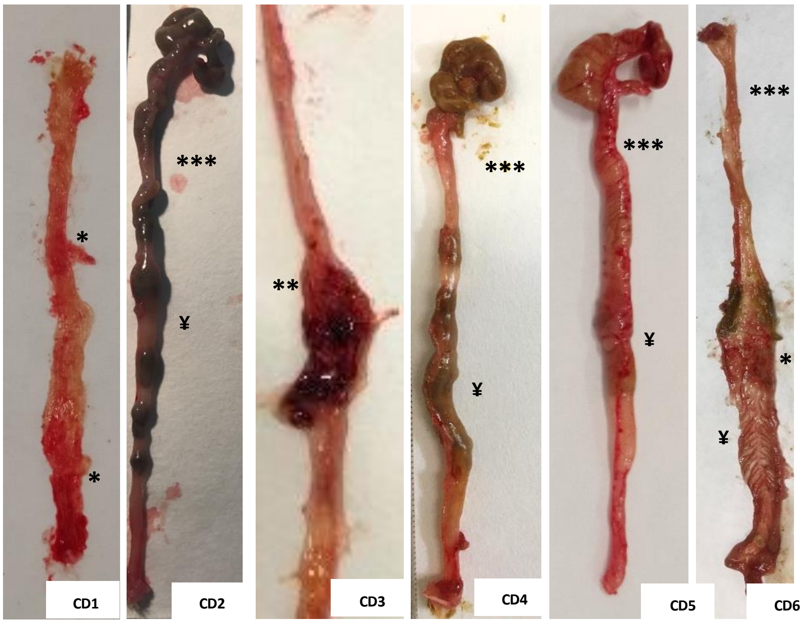

Crohn Disease (CD) group

Colon images from CD group (CD) of each mouse. Images show * Hyperaemia, ** Ulcer and/or perforation, *** shortening colon, ¥ oedema.

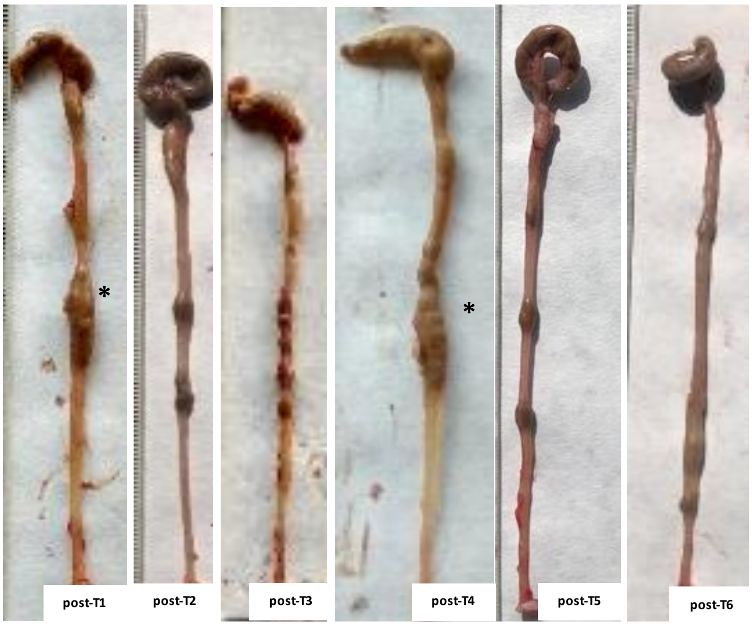

post-Treatment (post-T) group

Colon images from post-Treatment group (pre-T) of each mouse. Images show *slight oedema and absence of ulcer or any other colon tissue abnormalities.

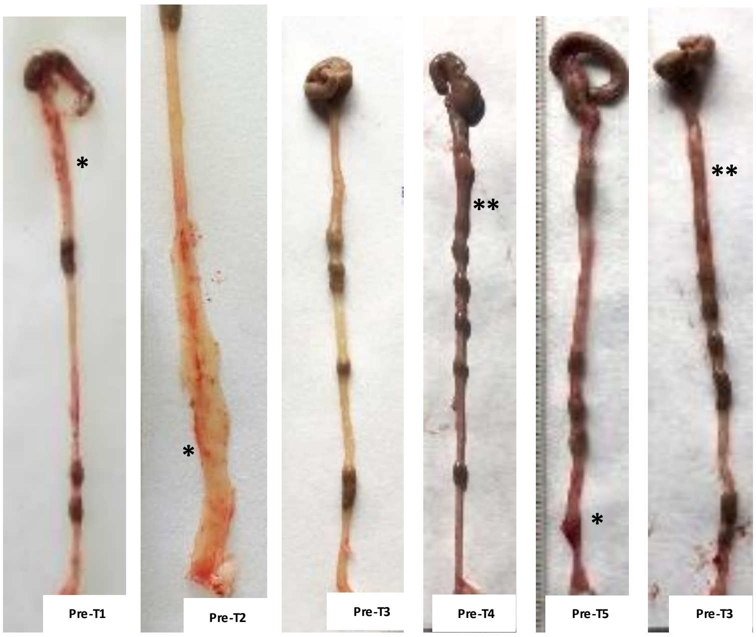

pre-Treatment (pre-T) group

Colon images from pre-Treatment group (pre-T) of each mouse. Images show * Slight hyperaemia, ** slight oedema and absence of ulcer or any other colon tissue abnormalities.
