# Supplementary material for: Effects of polyphenolic maqui (Aristotelia chilensis) extract on the inhibition of NLRP3 inflammasome and activation of mast cells in a mouse model of Crohn’s disease-like colitis
Source: Front Immunol. 2024 Jan 12;14:1229767. doi: 10.3389/fimmu.2023.1229767 (PMC10811055; doi:10.3389/fimmu.2023.1229767)
Supplement: Supplementary file 2 [file DataSheet_2.pdf]

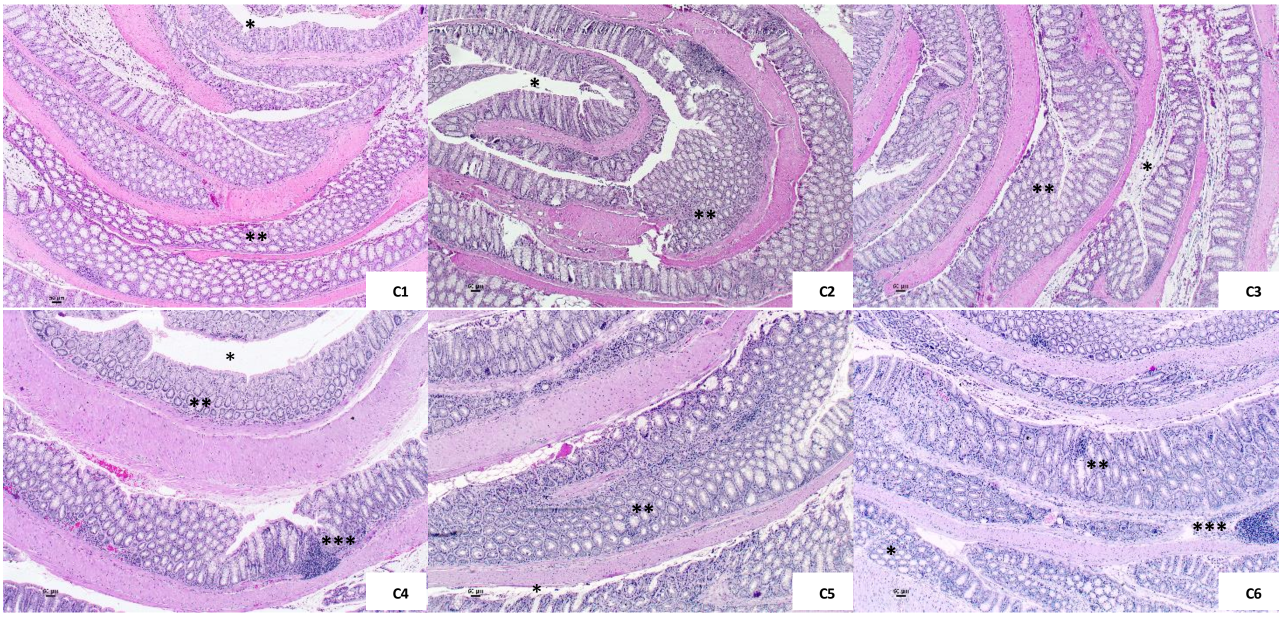

Control (C) group

Normal histoarchitecture in colon´s tissue from Control group (C) of each mouse. *Conserved mucosa, ** Normal crypts and, ***Physiological immune cell infiltration (lymph nodule). All images were captured at 40X

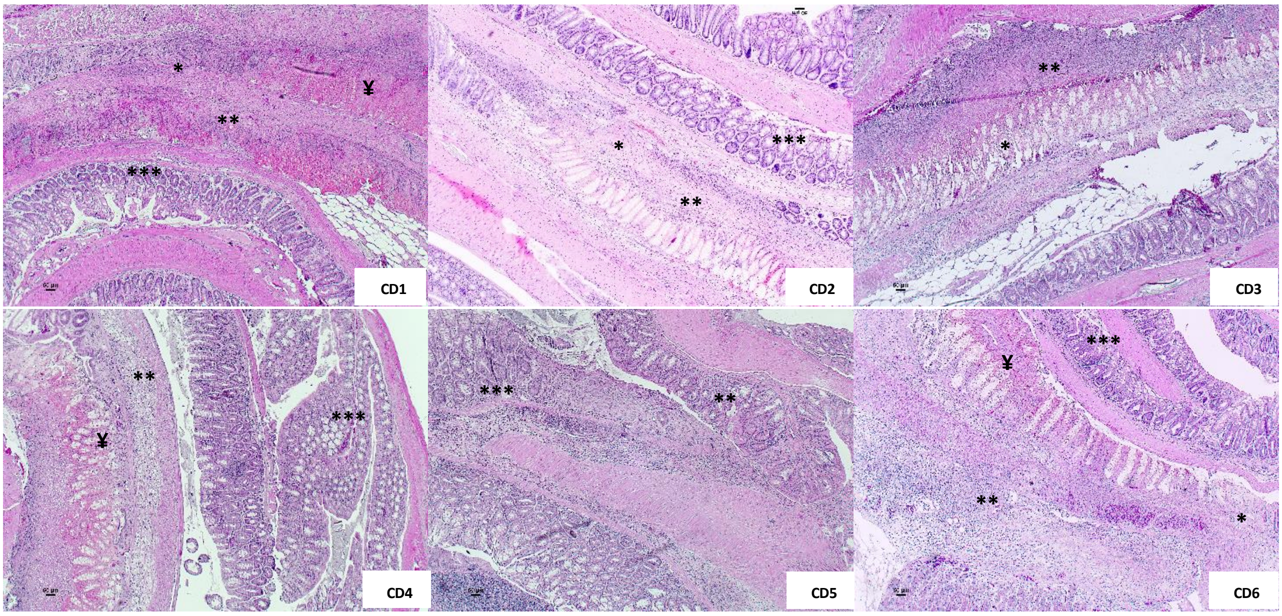

Crohn Disease (CD) group

Colon images from CD group (CD) of each mouse. *Distortion of colon architecture, ** Inflammatory cell infiltration, *** Loss of goblet cells and, ¥ Necrosis. All images were captured at 40X

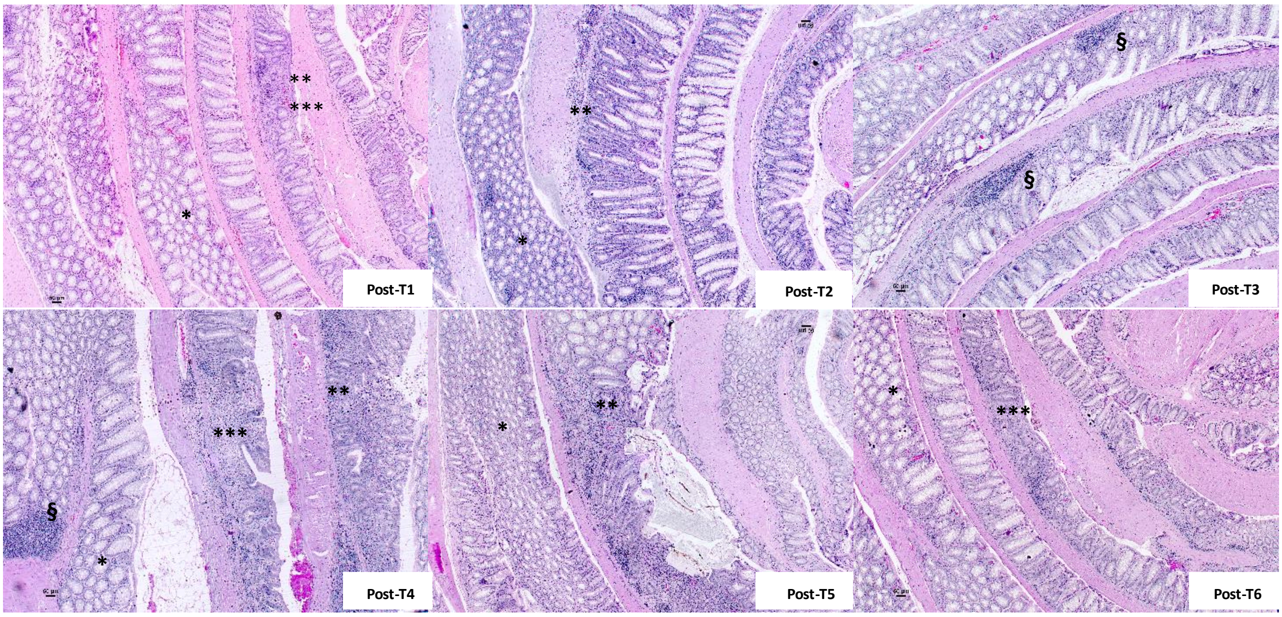

post-Treatment (post-T) group

Colon images from post-Treatment group (pre-T) of each mouse. * Normal histoarchitecture, ** Immune cell infiltration, ***Loss of crypts and, § Physiological immune cell infiltration (lymph nodule). All images were captured at 40X

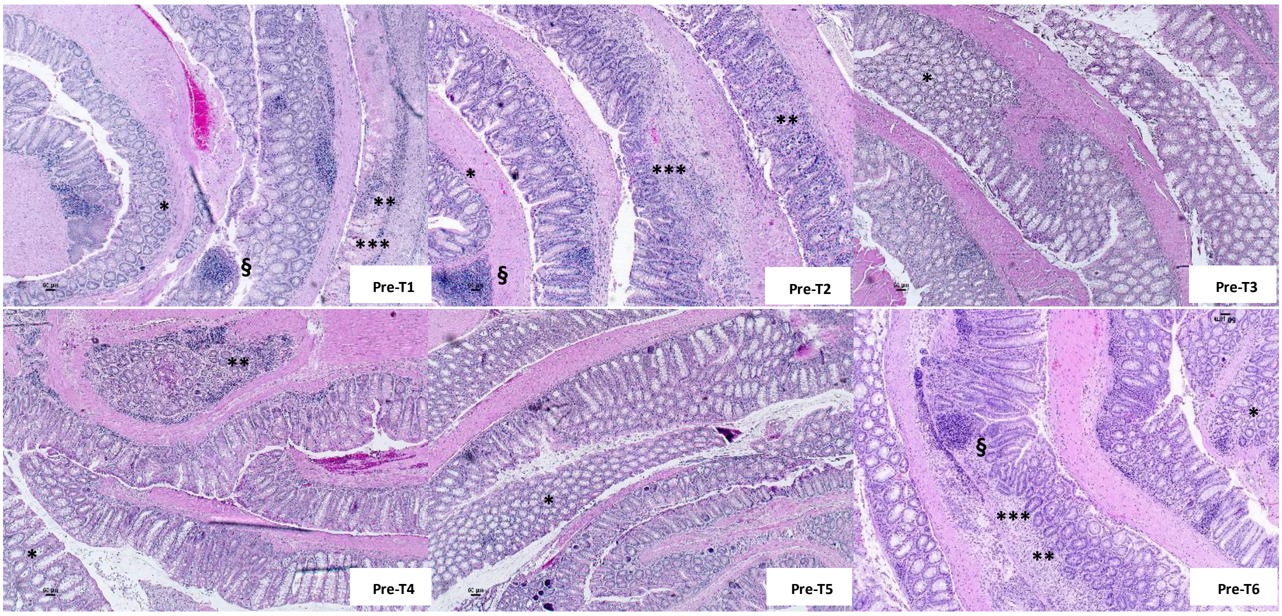

pre-Treatment (pre-T) group

Colon images from pre-Treatment group (pre-T) of each mouse. * Normal histoarchitecture, ** Immune cell infiltration, ***Loss of crypts and, § Physiological immune cell infiltration (lymph nodule). All images were captured at 40X.
